# High MELD score and extended operating time predict prolonged initial ICU stay after liver transplantation and influence the outcome

**DOI:** 10.1371/journal.pone.0174173

**Published:** 2017-03-20

**Authors:** Panagiota Stratigopoulou, Andreas Paul, Dieter P. Hoyer, Stylianos Kykalos, Fuat H. Saner, Georgios C. Sotiropoulos

**Affiliations:** Department of General, Visceral and Transplantation Surgery University Hospital Essen, Essen, Germany; Rutgers New Jersey Medical School, UNITED STATES

## Abstract

**Background:**

The aim of the present study is to determine the incidence of a prolonged (>3 days) initial ICU-stay after liver transplantation (LT) and to identify risk factors for it.

**Patients and methods:**

We retrospectively analyzed data of adult recipients who underwent deceased donor first-LT at the University Hospital Essen between 11/2003 and 07/2012 and showed a primary graft function.

**Results:**

Of the 374 recipients, 225 (60.16%) had prolonged ICU-stay. On univariate analysis, donor INR, high doses of vasopressors, “rescue-offer” grafts, being hospitalized at transplant, high urgency cases, labMELD, alcoholic cirrhosis, being on renal dialysis and length of surgery were associated with prolonged ICU-stay. After multivariate analysis, only the labMELD and the operation’s length were independently correlated with prolonged ICU-stay. Cut-off values for these variables were 19 and 293.5 min, respectively. Hospital stay was longer for patients with a prolonged initial ICU-stay (p<0.001). Survival rates differed significantly between the two groups at 3 months, 1-year and 5-years after LT (p<0.001).

**Conclusions:**

LabMELD and duration of LT were identified as independent predictors for prolonged ICU-stay after LT. Identification of recipients in need of longer ICU-stay could contribute to a more evidenced-based and cost-effective use of ICU facilities in transplant centers.

## Introduction

Liver transplantation (LT) represents a complex and challenging field whose settings has changed remarkably over the past few years. Since LT became a universally accepted treatment for end-stage liver disease (ESLD), the number of patients registered on the waiting list has gradually outweighed the scarce resources of available grafts. Implementation of Model for End Stage Liver Disease (MELD) score for allocation of deceased donor grafts both in USA [1] and within the Eurotransplant [2] aimed to reduce waiting list mortality and to prioritize candidates according to severity of liver disease. Moreover, the inadequacy of organ supply resulted in an expansion of donor/graft criteria.

In the context of increasing recipient disease severity and overall decreasing graft quality, there has been an incentive to identify predictors of outcome after LT. Numerous studies have analyzed the impact of recipient, donor and surgical characteristics on survival and efforts have been made to develop scoring systems for mortality after LT [3–6]. Fewer data exist with respect to the impact that these factors have on morbidity, as this is signified by length of intensive care unit (ICU)/hospital stay or the incidence of postoperative complications [7–10].

Over the past few years, optimal ICU management of liver recipients contributed to better outcomes after LT [11]. Expectations for the use of ICU facilities have changed remarkably. In the early 90s, postoperative mechanical ventilation for up to 36 hours was reported even for uncomplicated cases, resulting in a mean of 6-day ICU stay [12]. Although there have been attempts towards avoidance of ICU admission for selected patients [13–15], ICU management still remains part of the routine recovery process for the majority of LT recipients in Europe. Only limited data have been published on the factors that affect length of ICU stay after LT [7–8]. MELD score was the recipient characteristic more often associated with longer stay [7–8], but other recipient-specific variables have not been identified and the role of donor and surgical factors remain undefined.

Predicting length of ICU stay after LT could be very useful for both the transplant center and the party responsible for funding. Firstly, early identification of recipients in need of a prolonged ICU stay could allow the transplant team to plan ahead especially if restricted availability of ICU beds poses a limit to the evolution of the transplant program. Furthermore, efforts to modify factors contributing to prolonged ICU stay may potentially reduce the use of ICU facilities after LT and, in turn, the associated cost. The present study aimed to determine the incidence of prolonged initial ICU stay after LT and to identify recipient, donor, and surgical factors associated with it. The influence of prolonged ICU stay on patient/graft survival has also been investigated.

## Patients and methods

### Data collection

The medical records of adult patients who underwent LT between November 2003 and July 2012, at the University Hospital of Essen were reviewed. Patients were excluded if they survived less than 3 days after LT, if they were retransplanted, if they received multiple organs or living donor organ or if they were diagnosed with early allograft dysfunction (EAD) postoperatively. Donor data were obtained from the Eurotransplant International Foundation Database. This retrospective, single-centre cohort study was approved by the local ethics committee of the University Hospital Essen and followed the ethical guidelines of Declaration of Helsinki from 1975. The ethics committee waived informed consent because of the retrospective design.

Recipient information collected included age, gender, weight, height, body mass index (BMI), laboratory MELD score at transplant, indication for LT, “high urgency” listing, the presence of hepatocellular carcinoma (HCC), medical condition at the time of LT (at home, hospitalized, in the ICU), need for renal replacement therapy (RRT), the presence of diabetes mellitus. MELD was calculated according to United Network for Organ Sharing (UNOS) adjustments [16].

Donor data collected included age, gender, weight, height, BMI, cause of death, presence of diabetes mellitus, length of ICU stay, need for vasopressor support (no vasopressor, low <0.1μg/kg/min, moderate 0.1-0.5μg/kg/min, high >0.5μg/kg/min). Donor laboratory values recorded were SGOT, SGPT, INR, bilirubin, serum Na, creatinine. Graft information collected included graft type (split vs. whole organ), type of allocation (normal vs. rescue offer), graft quality as assessed by surgeons at the time of procurement (good, acceptable, poor), biopsy-proved steatosis (total, macrovesicular, microvesicular), preservation solution (University of Wisconsin, UW vs. Histidine-Tryptophane-Ketoglutarate, HTK), cold ischaemia time (CIT), donor risk index (DRI). Rescue organ offer was defined as a liver rejected by more than 3 different centers within the Eurotransplant due to medical, logistical or combined medical/logistical reasons [2]. CIT was defined as the interval from deceased donor cross-clamping to removal from storage for anastomosis. DRI was calculated using the formula constructed by Feng [17].

Transplantation information collected included duration of operation and warm ischaemia time (WIT). WIT was defined as the interval between removal of the graft from the preservation solution and venous reperfusion.

Posttransplantation information collected included length of initial ICU stay, length of hospital stay (LOS), presence of early allograft dysfunction (EAD). Patient/graft survival were calculated at 3 months, 1 year and 5 years after LT. Prolonged ICU stay was defined as an immediate stay in the ICU for more greater than 3 consecutive days after LT. EAD was defined as bilirubin ≥10mg/dl on 7th postoperative day and/or INR≥1.6 on 7th postoperative day and/or AST or ALT>2000IU/L within the first week [18].

### Statistical analysis

Data were collected using the ACCESS Database software (MS office 2003; Microsoft Corporation Redmond, WA, USA). Statistical analysis was performed using the SPSS Version 22.0 Software Package for Windows (IBM SPSS Statistics for Windows Version 22.0 Armock, NY; IBM Corp Released 2013, USA). Continuous variables are presented as means ± SDs as well as medians and interquartile range. Categorical variables were compared by chi-squared test. Recipient, donor and transplant variables were assessed in univariate Cox proportional hazards models to identify risk factors associated with prolonged initial ICU stay. Variables that were significant in univariate analyses (p<0.05) were subjected to multivariate analysis (stepwise fashion). Multivariate analysis was performed by logistic regression. Results from regression analysis were used to construct a prognostic score for prolonged ICU stay after LT. Its validity was tested using the area under the receiver operating characteristic (ROC) curve. For each continuous variable found significant in logistic regression model, a cutoff point was derived using the area under the ROC curve. Missing values were multiply imputed with Multivariate Imputation by Chained Equations. Patient/graft survival were determined by Kaplan-Meier analysis and compared with the log-rank test. P-value <0.05 was considered statistically significant.

## Results

### Study population

**Fig 1:**
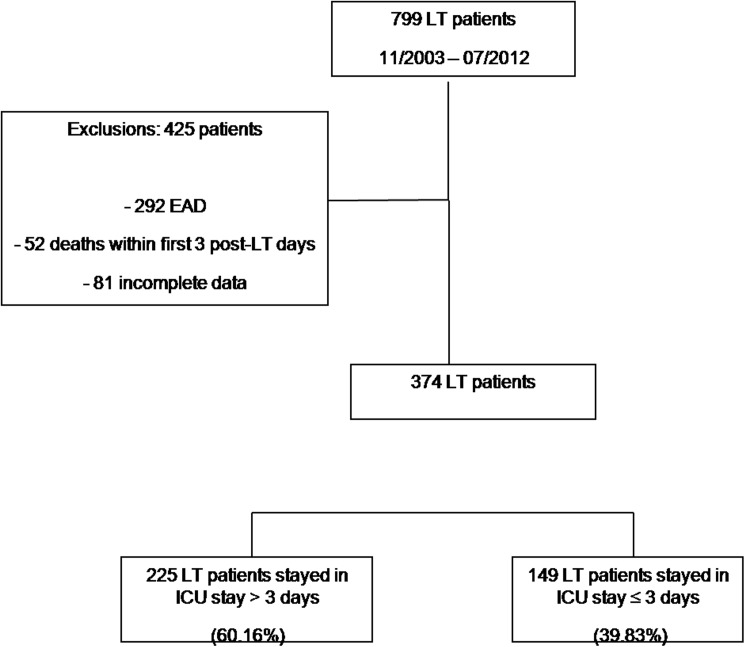
Flow diagram of patients included in the study.

During the study period (11/2003 –07/2012) 799 patients underwent LT in our center. Of these 799 patients, 344 were excluded according to the exclusion criteria and 81 were excluded due to incomplete data. Thus, a total of 374 recipients were entered for analysis (Fig 1).

### Recipient characteristics

The demographic and clinical characteristics of LT recipients are summarized in Table 1.

**Table 1:** Demographic and clinical characteristics of liver transplant recipients included in the study (n = 374).

Gender	
Male	224 (59.9%)
Female	150 (40.1%)
Age (years)	51.24 ±10.25
Height (m)	1.7279 ±0.12965
Weight (Kg)	79.41 ±16.82
BMI(Kg/m^2^)	26.44 ±4.87
Medical condition before LT	
At home	195 (52.2%)
Hospitalized	131 (35%)
In ICU	48 (12.8%)
‘high urgency’ listing	34 (9.1%)
Lab-MELD	19.32 ±9.59
Diabetes Mellitus	
Yes	77 (20.6%)
No	297 (79.4%)
Hepatocellular	
Yes	84 (22.5%)
No	290 (77.5%)
RRT before LT	
Yes	40 (32.8%)
No	82 (67.2%)
LT Etiology	
Alcoholic liver disease	101 (27%)
Acute hepatic failure	38 (10.2%)
Post-hepatic C Cirrhosis	100 (26.7%)
Post-hepatic B Cirrhosis	32 (8.6%)
Primary /Secondary sclerosing cholangitis	39 (10.4%)
Other causes (metabolic diseases etc)	64 (17.1%)

### Incidence of ICU stay / hospital mortality

Prolonged initial ICU stay (>3 days) was observed in 225 recipients (60.16%), whereas 149 patients (39.83%) were discharged from the ICU on days 1-3 after LT. The median length of initial ICU stay for the entire population was 4 days (1-127) vs. 7 days (4-127) for patients with a prolonged stay. The mean LOS was 29.87±19.73 days. LOS differed significantly between recipients with an ICU stay shorter than 3 days and those with a prolonged ICU stay (27.78±10.30 vs. 35.23±22.48, p<0.001). Median follow-up of the study was 3041 days.

Thirty-eight patients (10.16%) died before leaving hospital. Only one of them was discharged from the ICU earlier than the 3^rd^ post-transplant day. Thus, hospital mortality was significantly higher for patients who had a prolonged ICU stay than for those with a shorter ICU stay (p<0.001).

### Analysis for prognostic factors

Tables 2 and 3 provide results from the logistic regression analysis. In univariate analysis, only three donor/graft factors were found to be significantly associated with prolonged ICU stay: last donor INR (p = 0.044), high doses of vasopressors (p = 0.015) and “rescue offer” grafts (p<0.001) (Table 2). Five recipient characteristics were significantly associated with prolonged ICU stay: recipients hospitalized in the ICU before LT (p<0.001), candidates listed as high urgency cases (p = 0.002), labMELD at transplantation (p<0.001), alcoholic cirrhosis (p = 0.005) and need for RRT before LT (p = 0.001) (Table 2). Of the transplant characteristics, only the duration of operation (p = 0.005) was found to significantly correlate with prolonged ICU stay (Table 2).

**Table 2:** Univariate Cox proportional hazard regression analysis for prolonged ICU stay after LT.

	ICU ≤3 days	ICU >3 days	
	n = 149	n = 225	p-value
**Donor factors**			
Gender			0.399
Male	73 (49%)	100 (44.4%)	
Female	76 (51%)	125 (55.6%)	
Age (years)	55.68 ±19.23	51.82 ±18.97	0.057
Height (m)	1.702 ±0.173	1.7053±0.13	0.833
Weight (Kg)	77.47 ±19.20	75.04 ±16.52	0.193
BMI(kg/m^2^)	26.15 ±5.17	25.53 ±4.56	0.224
Cause of death			
Cerebrovascular Accident	94 (63.5%)	139 (62.1%)	
Trauma	22 (14.9%)	28 (12.5%)	
Anoxia	11 (7.4%)	35 (15.6%)	0.082
Other	21 (14.2%)	22 (9.8%)	
Steatosis micro (%)	20 (0-90)	10 (0-95)	0.059
Steatosis macro (%)	5 (0-80)	5 (0-50)	0.297
Graft quality as assessed by surgeon			
Good	105 (70.5%)	179 (79.6%)	
Acceptable	42 (28.2%)	42 (18.7%)	0.095
Poor	2 (1.3%)	4 (1.8%)	
γGT (U/L)	42 (4-717)	40 (6-766)	0.334
SGOT (U/L)	47 (9-6480)	48.5 (9-1299)	0.234
Serum Sodium (mmol/L)	147.22 ±8.45	147.62 ±8.51	0.654
SGPT (U/L)	32 (6-3272)	31 (4-1200)	0.546
Total bilirubin (μmol/L)	5.85 (0-116)	5.25 (0.08-145)	0.627
INR	1.155 (0.89-10.93)	1.14 (0.82-3.84)	0.044
Creatinine (μmol/L)	9 (0.10-129.86)	9 (0.11-88.21)	0.957
Rescue offer	110 (73.8%)	94 (41.8%)	<0.001
Perfusion solution			
HTK	107 (73.8%)	159 (76.4%)	0.616
UW	38 (26.2%)	49 (26.6%)	
Cold ischaemic time (min)	452 ±117.24	429 ±135.95	0.094
DRI	1.75 ±0.41	1.69 ±0.38	0.114
Diabetes Mellitus			
Yes	15 (10.1%)	16 (7.1%)	0.341
No	134 (89.9%)	209 (92.9%)	
Vasopressor requirement			
None	17 (12.6%)	28 (13.3%)	
Mild	51 (37.8%)	88 (41.9%)	
Moderate	44 (32.6%)	81 (38.6%)	
High	23 (17%)	13 (6.2%)	0.015
**Recipient factors**			
Gender			0.747
Male	91 (61.1%)	133 (59.1%)	
Female	58 (38.9%)	92 (40.9%)	
Age (years)	51.64 ±10.22	50.98 ±10.29	0.537
Height (m)	1.7282 ±0.09776	1.7277 ±0. 14723	0.973
Weight (kg)	77.72 ±14.81	80.53 ±17.98	0.114
BMI(k/m^2^)	25.98 ±4.11	26.75 ±5.3	0.139
Medical Condition before LT			
Not Hospitalized	88 (59.1%)	107 (47.6%)	
Hospitalized	56 (37.6%)	75 (33.3)	
ICU	5 (3.3%)	43 (19.1%)	<0.001
‘high urgency’ listing	5 (3.4%)	29 (12.9%)	0.002
Lab-MELD	14.43 ±6.83	22.56 ±9.79	<0.001
Diabetes Mellitus			
Yes	35 (23.5%)	42 (18.7%)	0.296
No	114 (76.5)	183 (81.3%)	
RRT before LT			
Yes	4 (10.8%)	36 (42.4%)	0.001
No	33 (89.2%)	49 (59.6%)	
LT Etiology			
Alcoholic liver disease	38 (25.5%)	63 (28%)	0.005
Acute hepatic failure	5 (3.3%)	33 (14.7%)	
Post-hepatic C Cirrhosis	49 (32.9%)	51 (22.6%)	
Post-hepatic B Cirrhosis	14 (9.4%)	18 (8%)	
Primary/Secondary sclerosing cholangitis	19 (12.8%)	20 (8.9%)	
Other causes	24 (16.1)	40 (17.8%)	
**Transplant procedure factors**			
Duration of operation (min)	269.55 ±70.76	296.24 ±86.98	0.005
Warm ischaemic time (min)	34.34 ±22.3	33.75 ±12.67	0.752

**Table 3:** Number of recipients in each MELD group.

Length of ICU stay	<10	11-19	20-29	>30	Total (n)
≤ 3 days	41 (27.50%)	80 (53.70%)	23 (15.40%)	5 (3.40%)	149
>3 days	22 (9.80%)	69 (30.70%)	71 (31.55%)	63 (28.0%)	225
Total (n)	63 (18.31%)	149 (39.83%)	94 (25.13%)	68 (18.18%)	374

**Fig 2:**
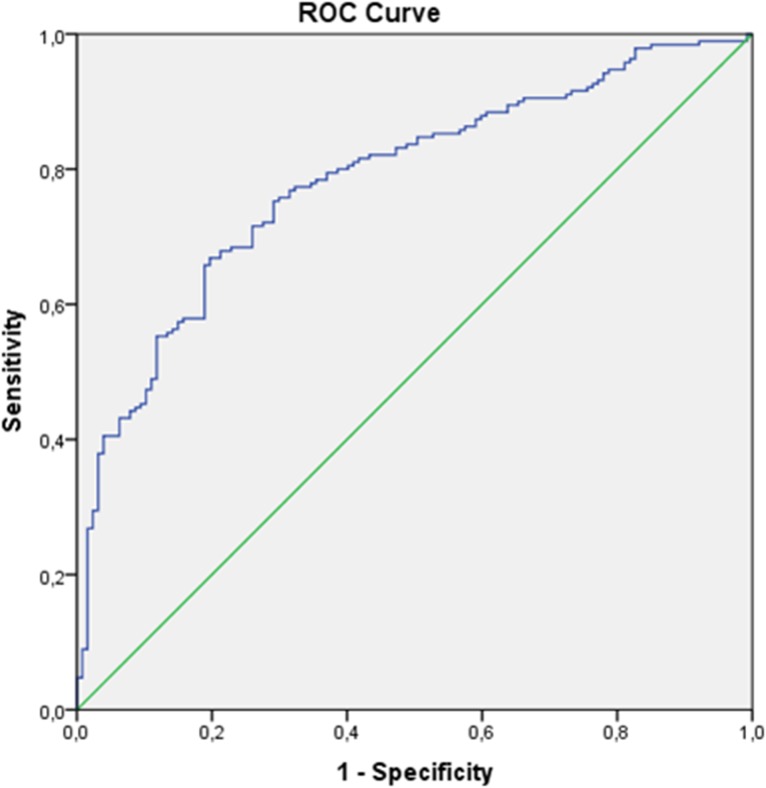
Receiver Operating Characteristic curve (ROC) curve associated with the logistic regression model.

Multiple regression analysis identified lab MELD (p<0.001, OR 1.122, 95%CI 1.088-1.157) and duration of operation (p = 0.009, OR 1.004, 95%CI 1.001-1.007) as the only parameters that independently correlated with a prolonged ICU stay. The Receiver Operating characteristic (ROC) curve associated with the logistic regression model is shown in Fig 2.

**Fig 3:**
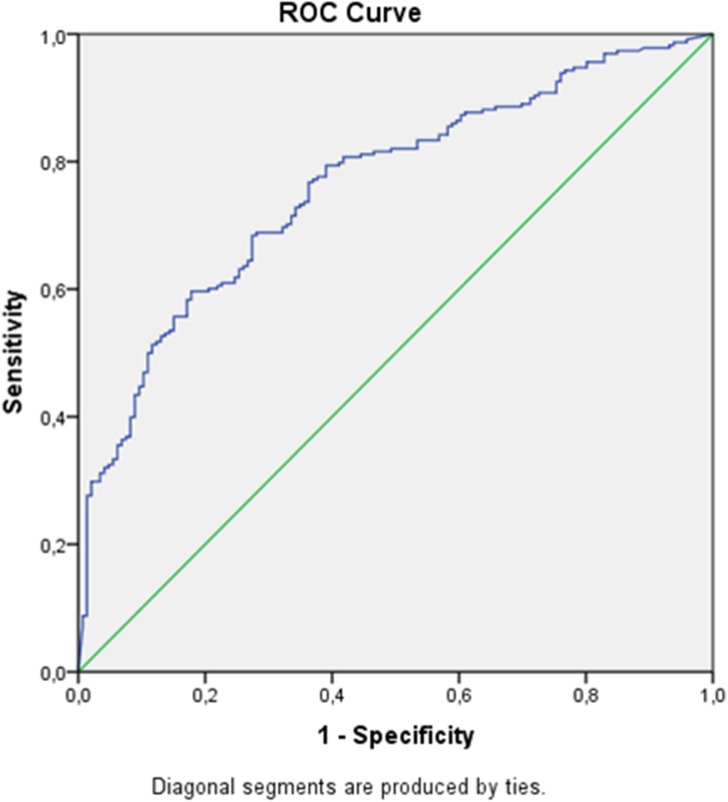
Receiver Operating Characteristic (ROC) curve for LabMELD.

The area under curve (AUC) is 0.784 [SE = 0.026 and 95% CI (0.734, 0.834), p<0.001]. Lab MELD is the overriding prognostic factor (in the univariate logistic model with LabMELD as the sole predictor, AUC = 0.762). The optimal cutoff point for Lab MELD is 19 and, in this case, the sensitivity of the model predictions is 78%, while the specificity is 67% (Fig 3).

**Fig 4:**
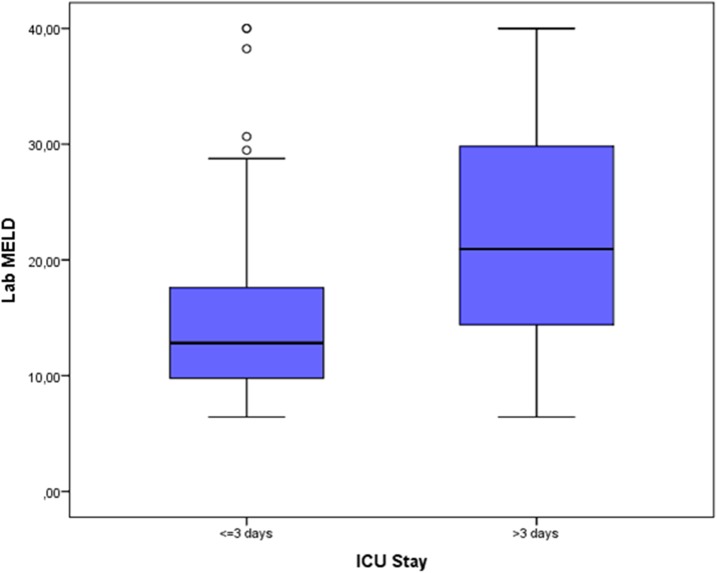
Box-Whisker-Plot depicting the influence of labMELD score on the length of ICU stay.

**Fig 5:**
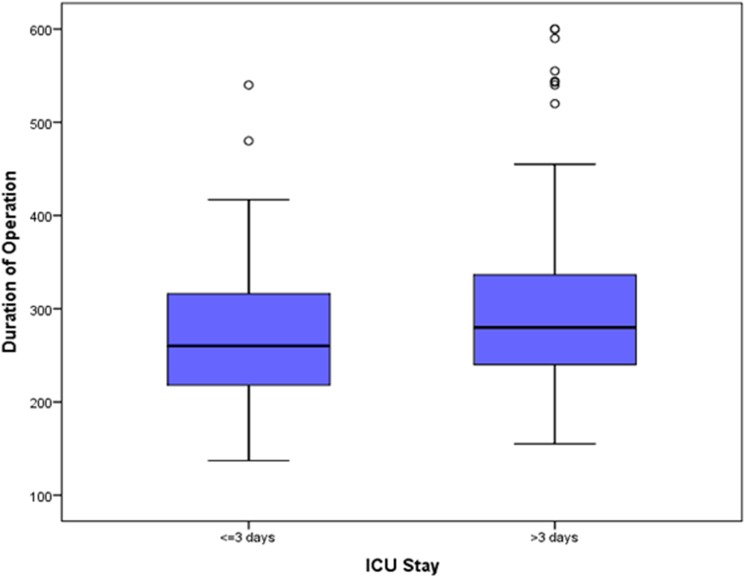
Box-Whisker-Plot depicting the influence of the duration of operation on the length of ICU stay.

The influence of labMELD score on the length of ICU stay is shown in Fig 4. Box-plot in Fig 5 shows the correlation of the length of surgery with the prolonged ICU stay after LT. A significantly higher labMELD and significantly longer surgery times were observed in the group of patients with a prolonged ICU stay.

The distribution of recipients into four categories according to their labMELD is shown in Table 3 (MELD: <10, 11-19, 20-29, ≥30). Of the 68 patients with MELD higher than 30, only 5 (7.40%) were discharged from the ICU within 3 days after LT. The majority of recipients with MELD >30 (63 patients, 92.60%) experienced a prolonged ICU stay. Similarly, of the 94 patients with MELD score between 20-29, only 23 (24.50%) had a short ICU stay. In contrast, in the lower MELD categories (<10, 11-19) MELD was not associated with a prolonged ICU stay.

Cutoff points were defined for whether a patient will have a prolonged stay in ICU: labMELD = 19, duration of operation = 293.5min.

### Predictive score for prolonged initial ICU stay

Based on the results of multivariable regression analysis, a formula has been computed indicating the probability of a recipient to stay in the ICU for more than 3 consecutive days after LT:

1/[1+EXP(-(-2.869+0.15×LabMELD+0.004×Duration of operation(min)))]

The cross-validated c-index for the original data set was 0.72555, indicating good discrimination.

### Patient- and graft-survival analyses

**Fig 6:**
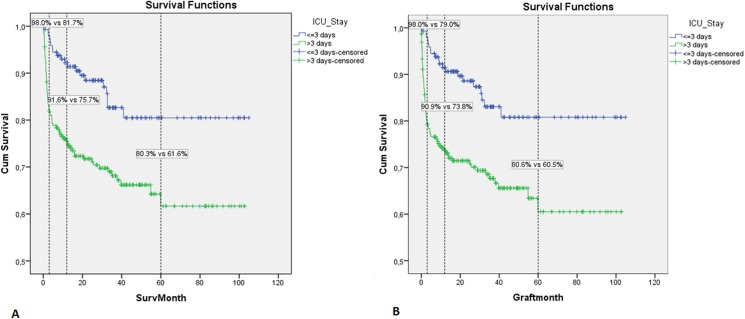
Kaplan-Meier survival analysis in LT recipients with and without a prolonged ICU stay after LT. A) 3 months, 12-months and 60-months patient-survival rates are shown. B) 3-month, 12- month and 60- month graft-survival rates are shown.

During the follow-up period, the overall mean patient-survival was 77.345 months (95%CI = 72.341-82.349). The mean survival of recipients with a prolonged ICU stay was significantly lower than that of patients discharged from the ICU within the first 3 post-transplantation days (69.057months, 95%CI = 62.402-75.711 vs. 87.943months 95%CI = 81.162–94.724, Log Rank test 14.088, p<0.001). Survival rates differed significantly between the two groups at all time points calculated (81.7% vs. 98% at 3 months, 75.7% vs. 91.6% at 1 year and 61.6% vs. 80.3% at 5 year, p<0.001) (Fig 6A). For the total cohort, the mean graft-survival rate was 76.825 months (95%CI = 71.699–81.951). Mean graft-survival for patients with a prolonged ICU stay was significantly lower compared to graft survival for patients with a prolonged ICU stay (67.959months, 95%CI = 61.058-74.861 vs. 88.0488 months, 95%CI = 81.289 -94.808, Log Rank test 15.458, p< 0.001). Differences between the two groups were obvious at all time points calculated (79.0% vs. 98% at 3 months, 73.8% vs. 90.9% at 1year and 73.8% vs. 90.9% at 5 year, p<0.001) (Fig 6B).

## Discussion

In the present study, we demonstrated that for LT recipients with satisfactory graft function, the labMELD at time of transplantation and the length of surgery were the two independent variables significantly associated with a prolonged initial ICU stay after LT. Furthermore, based on these variables, we created a prognostic score indicating the probability of a recipient to have a longer than 3 days ICU stay. To the best of our knowledge, this is the first study where such a prognostic score is constructed after analyzing recipient, donor, and procedural factors. Moreover, we clearly demonstrated that a prolonged initial ICU stay after LT is associated with prolonged total LOS, increased hospital mortality and impaired patient- and graft- survival.

Multivariate analysis revealed that higher MELD values immediately before LT significantly correlated with a prolonged ICU stay (HR = 1.122, 95% CI = 1.088-1.157, p<0.001). In particular, a MELD score increment of 1, increased the odds of a recipient to stay in the ICU for more than 3 consecutive days by 12.1%. MELD score was introduced as an allocation tool for deceased donor grafts first in the USA [1] and later in Europe (Eurotransplant 2006, Switzerland 2007) [2] with the primary aim to reduce mortality on the waiting list. Later, the impact of MELD score on variable end-points of LT outcome has been extensively analyzed. Our results regarding the potent effect of MELD score on prolonged ICU/hospital length of stay correlated well with 3 previous studies [7–9]. Washburn et al. [9] investigated the contribution of recipient/donor factors to LOS in two institutions and in the combined cohort. MELD was the only predictor present in all 3 cohorts with a strikingly similar effect: a MELD increment of 1 led to a 3% to 4% increase in LOS. Recently, Foxton et al. [7] reported that MELD score was significantly associated with a prolonged ICU stay, also defined as a stay greater than 3 days. In addition, Oberkofler et al. [8] identified MELD score as an independent risk factor for morbidity represented by an ICU stay longer than 10 days. The consistent effect of MELD score on the length of ICU/hospital stay in cohorts from 4 different centers, underlines the major effect of recipient disease severity on these outcome end-points.

As far as surgical factors are concerned, multivariate analysis demonstrated the duration of operation as an independent prognostic factor for prolonged initial ICU stay (HR = 1.004, 95% CI = 1.001-1.007, *p* = 0.009). We believe that the length of surgery alone offers estimation about the overall complexity of the transplantation procedure. It was noteworthy that in our cohort, if operating time increased by one minute, then the odds of the recipient to have a prolonged ICU stay increased by 0.4%. Since under the current allocation system labMELD score at transplantation might not be easily modified, the duration of the operation seems to be the only parameter that could be controlled, revealing a challenge for the transplant team to minimize operative time in order to improve outcome.

Interestingly, the warm ischaemic time (WIT) did not differ significantly between the two groups (33.75±12.67min vs. 34.34±22.3min, p = 0.752). WIT represents a technically complicated phase of the procedure and its duration depends mainly on surgeons’ experience. Long WIT in excess of 45 minutes was identified as a cause of irreversible graft injury [19]. However, no upper limit for acceptable WIT has been suggested yet, and given the fact that anastomotic times were similar for both groups in our cohort, we speculate that the complexity of the operation should lie on other aspects of intraoperative management. Thus, more information on the full spectrum of intraoperative course, including history of previous surgeries, anatomical variations, hemodynamic instability is needed and should be further analyzed so as to reveal possible causes for complicated transplantations and prolonged operative times.

Of the 374 patients included in our study, 225 recipients (60.16%) exhibited a prolonged initial ICU stay. This incidence was considerably higher than that reported by Foxton et al. [7] (136 patients out of the 402 stayed longer than 3 days in the ICU, 33.83%). Given the fact that only recipients with satisfactory graft function were included in our cohort, this finding deserves further explanation. One reason that may justify the higher incidence of prolonged ICU stay in our study could be the proportion of high-risk recipients. In particular, 68 patients (18.18%) had MELD >30 and the majority of them (63 patients, 92.60%) experienced a long post-LT ICU stay. In contrast, in the study of Foxton et al. [7] only 32 (8%) patients had MELD scores higher than 24, with 62.5% of them experiencing a prolonged ICU stay. Previous studies confirmed that recipients with MELD>30 form a subgroup with greater perioperative risks and expected delayed recovery compared to patients with lower MELD [20–22]. Characteristics that complicate perioperative management of high-MELD recipients are related not only to the variables included in the MELD formula, but also to MELD-unrelated factors (high vasopressor requirements, mechanical/vasopressor support pre-LT). Thus, the increased proportion of recipients with higher disease severity has contributed to the more complicated and prolonged recovery period after LT.

This is the first study that specifically addressed predictors for prolonged ICU stay exclusively for recipients with satisfactory graft function. Graft dysfunction is a well-recognized determinant of high morbidity/mortality after LT. Thus, we speculated that recipients with suboptimal graft function represented a subgroup of patients, which might bias our results. To address this issue, we separated the initial cohort into two groups: recipients with EAD and those without EAD, based on the definition by Olthoff et al. [18] The incidence of EAD in our cohort was 35.35%. Median length of ICU stay for patients with EAD was 6 days (1-161) in comparison to 4 days (1-127) for patients without EAD (data not shown, p = 0.0008). Furthermore, 1-year patient and graft survival differed significantly between the two groups (88.2%vs.73.9% and 82.0%vs.65.3%, p<0.0001, data not shown). Thus, the presence of EAD correlated not only with the dependent variable (length of ICU stay), but also with independent variables (graft/patient survival). This means that we had two different populations and it would be incorrect to treat them as one. Consequently, we decided to eliminate recipients with EAD from our analysis.

It is important to stress that in our study, no donor/graft variable emerged as independent predictor for a prolonged ICU stay after LT. Two reasons may explain this finding. Firstly, we limited our observation to the acute care setting i.e. we specifically focused on the immediate ICU stay after LT. Under these circumstances, it seems possible that the pre-LT recipient clinical profile has greater impact on the early clinical course in the ICU. Secondly, as it was previously mentioned, this is the first study that specifically identified predictors for prolonged ICU stay for recipients not diagnosed with EAD. Evidence exists that EAD can be predicted by donor/graft characteristics [17–23]. Thus, by excluding recipients who developed EAD, the influence of donor/graft variables was further restrained.

Our finding, regarding the absence of correlation between donor/graft characteristics and length of ICU stay is in consistent with two previous studies, indicating no correlation between graft quality as measured by the DRI and the total LOS [24] or an ICU stay >3 days [7]. These results are in contrast with the data of Axelrod et al. [25] who showed that the use of marginal grafts resulted in significantly increased LOS. Similarly, Oberkofler et al. [8] reported that the use of marginal grafts was a predictive factor for an ICU stay >10 days. These inconsistent results regarding the impact of donor/graft quality on ICU/hospital length of stay are in contrast with the consistent association of MELD with these variables in most of the studies previously mentioned. Factors such as single-center vs. multicenter (national) reports, as well as center-specific graft/recipient matching could potentially explain the inconsistent influence of donor variables on post-LT outcome parameters.

Another important finding was the correlation of a prolonged immediate ICU stay with impaired patient and graft survival, both in short-term and in long-term observation periods (98% vs. 81.7%, 91.6% vs. 75.7%, 80.3% vs. 61.6% at 3- 12- and 60-months for recipients with ≤3 days vs. >3 days ICU stay respectively, p<0.001). Smith et al. [10] reported inferior graft/patient survival for recipients with a LOS >30 days. To the best of our knowledge, our study is the first in the literature that showed a correlation between the initial ICU length of stay and survival after LT. This finding emphasizes the impact of the immediate recovery period on long-term LT outcome and thus, the importance of the early ICU management of LT recipients.

A successful prediction of post-LT ICU stay is of particular importance for centers where reduced availability of ICU beds or lack of ICU facilities dedicated to the transplant program may act as a limiting factor in their activity. In this context, predicting length of ICU stay could lead to a more efficient allocation and evidence-based use of ICU beds. Finally, successful modification of factors leading to prolonged ICU stay after LT may potentially reduce the use of hospital resources and, in turn, the overall cost. Most of the total cost after LT is spent on special care units [26] and the pressure to reduce cost seems to be even higher after the implementation of the MELD-based allocation system, since transplantation of sicker patients was translated to longer LOS and increased costs [27–31].

Our study is not without limitations, the most important being its retrospective single-center design. Thus, it is not guaranteed that the same risk factors would equally apply in other institutions with different recipient/donor characteristics and/or clinical practices regarding the perioperative care of LT recipients. Finally, our study is further limited by the lack of information about other aspects of intraoperative care, which could potentially affect the immediate ICU course of LT recipients.

In conclusion, in our cohort, the labMELD at the time of transplantation and the length of surgery were the two independent risk factors for an ICU stay longer than 3 days after LT. A prolonged initial ICU stay was strongly associated with longer LOS, higher hospital mortality and lower survival rates. The ability to identify patients in need of longer ICU stay is a valuable asset that could contribute to a more evidenced-based and cost-effective use of ICU facilities in transplant centers.

## Supporting information

S1 File Patients’ data. The S1_File.xls contains all data from our study population.
(XLS)
